# Sciatica

**Published:** 1893-06

**Authors:** Chas. B. Gilbert

**Affiliations:** Washington, D. C.


### SCIATICA.

Chas. B. Gilbert, M. D., Washington, D. C.

The following symptoms have been verified: none of the remedies were given low, especially Kali-bichrom.

Arnica: Hip sore as if bruised; bed feels hard. (I prefer Arnica-radix, but it acts so sharply, even in the thirtieth, that it needs to be watched.)

Kali-bich.: Aching in back and down left side into hip. Rheumatic pains in hip joints and knees on moving; on walking. Pain in the course of the left sciatic nerve from behind the great trochanter to the calf. (Hering's *Condensed.*) (The sciatic pains are apt to follow a cold in the head, by metastasis.)

Silicea: Right-sided sciatica better on motion and from heat; worse when at rest or when cold; costive.

(Rhus-tox. will not cure when the bowels are costive.)

Thuja: Pain in right sciatic nerve—when rising, first starting to walk, bending forward (putting on stockings), tired from standing at a desk: gonorrhœal history.

